# The *SAP* Gene Family in Oat (*Avena sativa* L.): Genome-Wide Identification, Gene Expression Analysis, and Functional Characterization of AvSAP1 in Response to Stress Conditions

**DOI:** 10.3390/life16010046

**Published:** 2025-12-26

**Authors:** Nour Regaig, Mouna Ghorbel, Ikram Zribi, Olfa Jrad, Kaouthar Feki, Khaled Masmoudi, Faiçal Brini

**Affiliations:** 1 Biotechnology and Plant Improvement Laboratory, Centre of Biotechnology of Sfax, University of Sfax, P.O. Box 1177, Sfax City 3018, Tunisia; 2 Department of Biology, College of Sciences, University of Hail, P.O. Box 2440, Ha’il City 81451, Saudi Arabia; 3 Integrative Agriculture Department, College of Agriculture and Veterinary Medicine, United Arab Emirates University, Al Ain P.O. Box 15551, United Arab Emirates; khaledmasmoudi@uaeu.ac.ae

**Keywords:** *Avena sativa*, abiotic stress, bioinformatics analysis, gene expression, recombinant protein, stress-associated proteins (SAPs)

## Abstract

The stress-associated proteins (SAPs) correspond to zinc-finger proteins containing A20/AN1 domains that are involved in plant responses to a wide range of biotic and abiotic stresses. However, in oat, no information has been available so far regarding the characteristics and regulation of these genes. In the current research work, eleven *AvSAP* genes were identified in oats genome (OT3098 variety) named *AvSAP1* to *AvSAP11*. Eight proteins contained both A20 and AN1 domains located at the N- and C-terminal portions of the proteins, respectively. Subsequently, the gene structure and duplication, chromosomal location, cis-acting elements, and protein properties were analyzed using bioinformatic tools. Moreover, genes expression profiles revealed that *AvSAP* genes present hormones and stress-responsive cis-elements in their promoters. These observations were confirmed using QRT-PCR analysis. Indeed, the majority of identified *AvSAP* genes were responsive to NaCl, PEG, heat, ethylene, and metallic (Mn, Cu, and Cd) stresses. Moreover, ABA phytohormone induced a significant upregulation of nine *AvSAP* genes in leaves (5.8–6.5-fold induction) and roots (1.9–4.2-fold induction), corroborating their crucial role of those genes in plants’ response to a wide range of abiotic stresses. In contrast, GA and IAA exert a slight effect on those genes. Finally, *AvSAP1* protein expression in bacterial cells conferred tolerance to ionic and osmotic stress. Our results provide deeper insight into *AvSAP* genes in plants and support advanced functional analyses of this gene family in oats.

## 1. Introduction

Various environmental stresses pose serious dangers to global agricultural crops, which, in fact, threaten food security. These stresses include extreme temperatures, drought, nutrient deficiency, salt, and exposure to toxic metals, and notably affect plant growth and productivity. Basically, these constraints lead to a decline in crop production worldwide by about 30–50% [1]. In addition, global climate change will worsen this situation in the coming years, raising a true challenge for governments to feed about 9 billion people by 2050, unless food production increases by about 70% [1,2]. From this perspective, opting for stress-tolerant crops is becoming essential to address this challenge [3].

In plants, the stress-associated proteins (SAPs) constitute a novel subclass of zinc-finger protein. Identified for the first time in rice, these proteins are structurally conserved in all investigated plant species [4,5] and display a combination of A20, AN1, and C2H2 domains. *A20/AN1 genes* are now considered to be the master regulators of plant tolerance to environmental constraints [4,5,6].

The A20 zinc-finger domain is formed by different Cys2-Cys2-finger motifs, while the AN1 zinc-finger domain contains multiple C2H2 finger motifs. Both domains may coexist in the same protein, separated by a variable stretch of amino acids. Some SAPs proteins also equally contain other motifs (C2H2, also known as Cys2-His2 RING motifs) at the C-terminus portion of the protein. This gene family has been shown to provide tolerance to diverse abiotic constraints [4,5,7,8,9,10,11,12,13,14]. In *Arabidopsis thaliana*, there are 14 *SAP* encoding genes containing A20, AN1, and Cys2-His2 zinc-finger domains. Overexpression of *AtSAP5* in cotton also promotes tolerance to water deficit and heat stress [15]. In addition, AtSAP13, an SAP protein presenting two AN1 zinc-finger domains and an additional C2H2 domain, boosts *Arabidopsis* plant tolerance to diverse abiotic stresses [16]. The constitutive expression of *SAP* genes in tobacco, *Arabidopsis*, banana, cotton, rice, and wheat is proven to positively regulate plant tolerance to different abiotic stresses. In this respect, transgenic rice and tobacco, overexpressing *OsiSAP1* and *OsiSAP8*, harboring one AN1 and one A20 domain each, display an enhanced tolerance to drought, salt, and cold [8,9,17]. In addition, tobacco plants overexpressing *OsiSAP1* genes equally exhibit an enhanced disease resistance against virulent bacterial pathogen and upregulated defense-related genes [18]. In Cassava, 11 *SAP* genes were identified [6], including three intron-free genes (Metip4, Metip8, and Metip11). Each of these three proteins presents one A20 and one AN1 domain, with considerable amino acid sequence variations of these domains in Metip4, Metip8, and Metip11 proteins. Metip4, Metip8, and Metip11 are nuclear proteins that do not interact with each other but act as positive regulators of plant responses to drought, salt, and extreme temperatures [10,19]. Additionally, these proteins play a positive role in plant responses to Mn stress [12].

A20/ANA1 proteins can be categorized into different taxonomic groups according to the number, type, and organization of their domains. This classification can be observed at both intra- and interspecies levels [4,19]. *SAP* genes can contain introns [5,20,21]. Moreover, some other *SAP* genes may lose their function during evolution [22]. Other analyses revealed the presence of 18 *SAP* genes in *Oryza sativa* genome [10], 17 in *M. truncatula* [23], 30 in the apple genome [13], 37 in cotton (*Gossypium hirsutum*) [24], 11 in Jatropha and *Tamarix hispida* [14], 10 in maize [21], 60, 16, 13, and 10 genes in *T. aestivum*, *Ae. tauschii*, *T. dicoccoides*, and *T. urartu*, respectively [25], 27 in sunflower (*Helianthus annuus*) [26], 19 in poplar [27], 13 in tomato [28], and 12 in cucumber [29].

Oats (*Avena sativa* L.), a vital and well-established cereal crop, are cultivated in many regions such as North America and Europe [30], as well as around the Mediterranean, the Middle East, the Canary Islands, and the Himalayas [31]. This crop adapts to different soil types and exhibits significant genetic diversity, as it may be found as diploids, tetraploids, and hexaploids [31,32]. It is noteworthy that this crop is frequently grown in regions with harsh conditions, such as drought or high salinity, as it displays greater tolerance compared to soybean, maize, and wheat crops [33].

The stress-adaptive mechanisms in oats at the biochemical and molecular levels remain largely obscure and have been scarcely addressed. Different research works have been conducted to investigate different genes implicated in oat plant responses to abiotic stresses such as WRKY [34], NAC [35], CAMTA [36], SOD [37], CAT [38], POD [39], aquaporine [40], and others. To our knowledge, despite their importance in plant responses to environmental stresses, no data are available on the SAP gene family in oats. In the present study, eleven *AvSAP* genes were identified and characterized from the oats (OT3098 variety) genome. Thus, phylogenetic relationships, proteins properties/locations, conserved domains, genes structures/duplications, cis-acting elements, and expression profiles in different tissues and under different environmental conditions were performed, revealing important modifications in response to different abiotic and hormonal stresses. Moreover, recombinant AvSAP1 proteins expressed in *E. coli* conferred abiotic stress tolerance to bacteria. This study is valuable and promising as it provides key insights into the underlying functions of *AvSAP* genes and may, therefore, help in exploring the known modes of *SAP* actions in plants. The obtained findings may serve as an enlightening guideline to trace the evolutionary history and biological functions of *AvSAP* genes in oats. This study also paves the way for further fruitful lines of investigation in terms of the role of *SAP* gene families in plants.

## 2. Materials and Methods

### 2.1. Identification of AvSAP Family Members

The conserved domain sequences of the A20 zinc-finger (Pfam ID: PF01754) and AN1 zinc-finger (Pfam ID: PF01428) served as queries for a BLAST 2.16.0 search against the *Avena sativa* genome (OT3098) variety) via EnsemblPlants database (https://plants.ensembl.org; accessed on 9 December 2024). From this database, all relevant nucleotide, protein, and GFF3 files were downloaded. Candidate AvSAP proteins with an E-value threshold of ≤10^−5^ were selected. To confirm the presence of A20 and/or AN1 zinc-finger domains, these candidate proteins were analyzed using InterPro database (http://www.ebi.ac.uk/interpro/; accessed on 9 December 2024), the NCBI Conserved Domain Database (https://www.ncbi.nlm.nih.gov/Structure/cdd/wrpsb.cgi; accessed on 9 December 2024), and SMART (http://smart.embl-heidelberg.de/smart/; accessed on 9 December 2024).

### 2.2. Physicochemical Properties and Structural Characterization of AvSAP Proteins

The physicochemical properties of AvSAP proteins were assessed using the ExPASy ProtParam tool (http://web.expasy.org/protparam/; accessed on 20 November 2024). To predict their subcellular localization, we used the WoLF PSORT online tool (https://wolfpsort.hgc.jp/; accessed on 14 December 2024). The three-dimensional structures of AvSAP proteins were modeled with the SWISS-MODEL server (https://swissmodel.expasy.org/interactive; accessed on 14 December 2024). The built models were validated by the ProSA-web online tool (https://prosa.services.came.sbg.ac.at/prosa.php; accessed on 10 October 2025) (Supplementary Table S1). The active site pockets in SAP structure of *A. sativa* were predicted by CASTp (Computed Atlas of Surface Topography of proteins) online server (http://sts.bioe.uic.edu/castp/calculation.html; accessed on 16 December 2024).

### 2.3. Analysis of Gene Structures, Localization, Duplication, and Their Promoter Regions

GFF3 files were used to visualize the exon–intron arrangements of *AvSAP* genes. Structural organization and chromosomal distribution of *Avena sativa SAP* (*AvSAP*) genes were analyzed using TBtools II v2.127. To identify the duplication gene events, protein and CDS sequences were invested to compute the non-synonymous and synonymous mutation values (Ka/Ks) in the software Tbtools II v2.127. The promoter regions located upstream of the initiation codon of each candidate gene were used to analyze their cis-acting regulatory elements with PlantCare database (https://bioinformatics.psb.ugent.be/webtools/plantcare/html/, accessed on 18 December 2024).

### 2.4. Evolutionary Relationships of AvSAP Family Members

Multiple alignments of *Avena sativa* SAP sequences were carried out using the CLUSTALW algorithm in the software MEGA 11. The aligned protein sequences were subsequently visualized applying GeneDoc 2.6.02 software. To analyze the relationship among SAP protein members, the neighbor-joining test was used to generate the phylogenetic trees with 1000 bootstrap replications. Additionally, different AvSAP protein orthologues from other plant species such as *Triticum aestivum*, *Oryza sativa*, and *Arabidopsis thaliana* were deployed.

### 2.5. RNA Extraction and Real-Time Quantitative PCR

Seeds of *Avena sativa* (Jebiniana, Sfax, Tunisia; obtained from INRA, Tunis, Tunisia) were surface-sterilized with 2.4% NaClO (sodium hypochlorite) for 12 min and washed multiple times with distilled water. Sterilized seeds were placed on wet filter papers in Petri dishes at room temperature (~25 °C) for 2 days to germinate, then grown in a growth chamber at 23 °C (day) and 15 °C (night) with a 16/8 h light/dark cycle and 50% humidity until the 10-day stage. At this stage, the seedlings were subjected to stress treatments with 150 mM NaCl, PEG (10%), cold (4 °C), heat (37 °C), hormonal treatments using 5 mM of six different hormones (abscisic acid (ABA), gibberellic acid (GA), auxin (AIA), salicylic acid (SA), jasmonic acid (JA), and etephon (Eth)), or heavy metal stress (500 µM ZnSO_4_, 50 µM CuSO_4_, 1 mM MnCl_2_, and 100 µM CdCl_2_. Roots and leaves samples were collected independently (~0.5 g of each tissue) at 0 h and 48 h post-treatment to assess the stress responses. Control samples were grown without stress. The samples were immediately frozen in liquid nitrogen, and stored at −80 °C. RNA was extracted using the RNeasy Plant Mini Kit (QIAGEN, Hilden, Germany) and reverse transcription was performed with the PrimeScript RT reagent Kit (TaKaRa) using 1 µg RNA. Gene-specific primers were designed with Beacon Designer 7.9 (Supplementary Table S2). The qRT-PCR reactions were performed using SYBR premix ExTaq II (TaKaRa) in 10 μL of final volume PCR reactions. The thermal cycling conditions were maintained as follows: 95 °C for 30 s followed by 40 cycles of 95 °C for 15 s, and 62 °C for 20 s. Data were analyzed using the 2^−ΔΔCT^ method, where ΔΔCT = (CT, target gene: CT, reference gene) stressed (CT, target gene: CT, reference gene) control. Each sample was subjected to three technical repeats and three biological repetitions for every stress level. Two different internal genes, namely, *ADP-ribosylation factor proteins* (*ADPR*) and *Glyceraldehyde-3-phosphate dehydrogenase* (*GAPDH*) (Supplementary Table S2), were used as internal expression standards, as stated previously [37,38,41].

### 2.6. Molecular Cloning of AvSAP1 cDNA

Using the TRIzol reagent (Invitrogen, Thermo Fisher Scientific, Waltham, MA, USA), total RNAs were extracted from the leaves of oat plants (10 days old) that had been exposed to 150 mM of NaCl for 24 h. The isolated RNA was processed with RNase-free DNase to remove the remaining genomic DNA. The synthesized CDSs were subsequently used for amplification through RT-PCR with gene-specific primers (Supplementary Table S3). PCR was subjected to 35 cycles, beginning with an initial denaturation at 95 °C for 2 min, followed by a cycle of denaturation at 95 °C for 30 s, annealing at 55 °C for 30 s, and extending at 72 °C for 1 min, concluding with a final extension step at 72 °C for 7 min. The resulting PCR products were purified using the QIAquick PCR Purification Kit (Qiagen, Hilden, Germany) and ligated into a pGEMT-Easy vector (Promega, Madison, WI, USA) by means of the NEB PCR Cloning Kit (New England Biolabs, Royaume Uni., Ipswich, MA, USA). Recombinant plasmid DNA was extracted from transformed *E. coli* cells and sequenced in both directions to confirm the sequence identity of the *AvSAP1* gene. After verification, the sequence of the newly isolated *SAP* gene was deposited in NCBI (accession number PX394204).

### 2.7. Production and Purification of Recombinant AvSAP1 Proteins

The product was amplified by PCR through the use of the Pfu Taq DNA polymerase and the proper primers containing *EcoR*I restriction sites (Supplementary Table S3), in order to create the recombinant proteins His_AvSAP1. The product was then digested by the proper restriction enzymes, *EcoR*I/*Not*I, and cloned in-frame with a Histidine-tag into the pET28a expression vector (Novagen, Madison, WI, USA). The resulting material (pHis_AvSAP1) was inserted into the Novagen, Pecs, Hungary, BL21 *E. coli* strain (DE3). Among colonies cultivated in solid LB medium containing 100 mg/mL Kanamycin, a single colony was selected then cultivated overnight at 37 °C. The culture was then grown to an OD of 0.6 at 600 nm and diluted 1:100 into new LB-Kanamycin media. Next, 1 mM of isopropyl-D-thiogalacto-pyranoside (IPTG) was incorporated into the cells overnight at 37 °C to induce SAP protein expression. Transformed bacteria were centrifuged at 4500 rpm for 10 min at 4 °C, and pellets were washed twice using cold water. The cells were then taken out and sonicated on ice in cold lysis buffer (Tris–HCl 100 mM pH 8; EDTA 1 mM; NaCl 120 mM; 1 mM DTT; 50 mM PMSF; and 0.5% Tween). Subsequently, cells were centrifuged for 45 min at 4 °C using a speed of 9000 rpm. SDS page analyses revealed that the supernatant did not contain the recombinant pHis_AvSAP1 protein. Hence, the recovered inclusion bodies, stored at (−20 °C), were resuspended and treated in the lysis buffer for an overnight period then centrifuged at 9000 rpm for 10 min at 4 °C. After preadjusting with binding buffer (Tris–HCl 100 mM pH 8; NaCl 0.5 M; 30 mM imidazole) and loading the supernatant onto a Ni-Sepharose column (Bio-Rad, Hercules, CA, USA), the supernatant was gravity-eluted. The Bradford technique was applied for protein quantification [42].

### 2.8. SDS-PAGE and Western Blot Analysis

Protein samples (5 μg each) were separated on a 10% SDS-PAGE gel alongside a protein molecular marker (Thermo Scientific™ PageRuler™ Prestained Protein Ladder, Waltham, MA, USA). The protein bands were either stained with Coomassie blue for visualization or transferred onto a nitrocellulose membrane (Schleicher and Schuell, BioScience GmbH, Dassel, Germany) using a semi-dry blotting system (Bio-Rad). To prevent non-specific binding, the membrane was blocked with PBST (10× PBS containing Tween 20 at a 1:1000 dilution) supplemented with 5% non-fat dry milk. The blot was incubated afterwards with a peroxidase-conjugated anti-His tag antibody (Abcam, Concord, ON, Canada) at a 1:2000 dilution in PBST containing 1% non-fat dry milk.

### 2.9. Expression of AvSAP1 Protein in E. coli and Stress Tolerance Assay

To assess the role of *AvSAP1* in *E. coli* stress tolerance, the recombinant vector pHis_AvSAP1 was introduced into the BL21 strain of *E. coli*. This strain was transformed with the empty vector in parallel. For stress tolerance assays, aliquots (5 μL) from the saturated *E. coli* cultures and tenfold serial dilutions (10^−2^, 10^−3^, 10^−4^, and 10^−5^) were spotted onto LB medium without (control) or with NaCl (200 mM), LiCl (500 mM), KCl (500 mM), PEG6000 (10%), and mannitol (150 mM). Colonies were photographed after 24 h of incubation at 37 °C.

### 2.10. Statistical Analysis

A two-way ANOVA was used separately for each variable (plant tissue, type of stress, and time after stress application) to compare the differences between stressed and non-stressed plants. Tukey’s pairwise comparison tests were conducted thereafter (as the data followed a normal distribution), with a significance level of *α* = 0.05 relative to the control group of untreated plants.

## 3. Results

### 3.1. Identification of Analysis of AvSAP Genes in Avena sativa Genome

*AvSAP* genes were comprehensively searched from the genome of the hexaploid oats (*Avena sativa*, OT3098 variety) via the Ensembl plants database. Redundant sequences were eliminated manually. At this stage, we identified 11 *AvSAP* genes (named *AvSAP1-11*). All these proteins harbor the conserved domain PF00304 (gamma-thionin). Gene sequences lengths ranged from 1385 to 3510 base pairs (bp) (Table 1).

**Figure 1:**
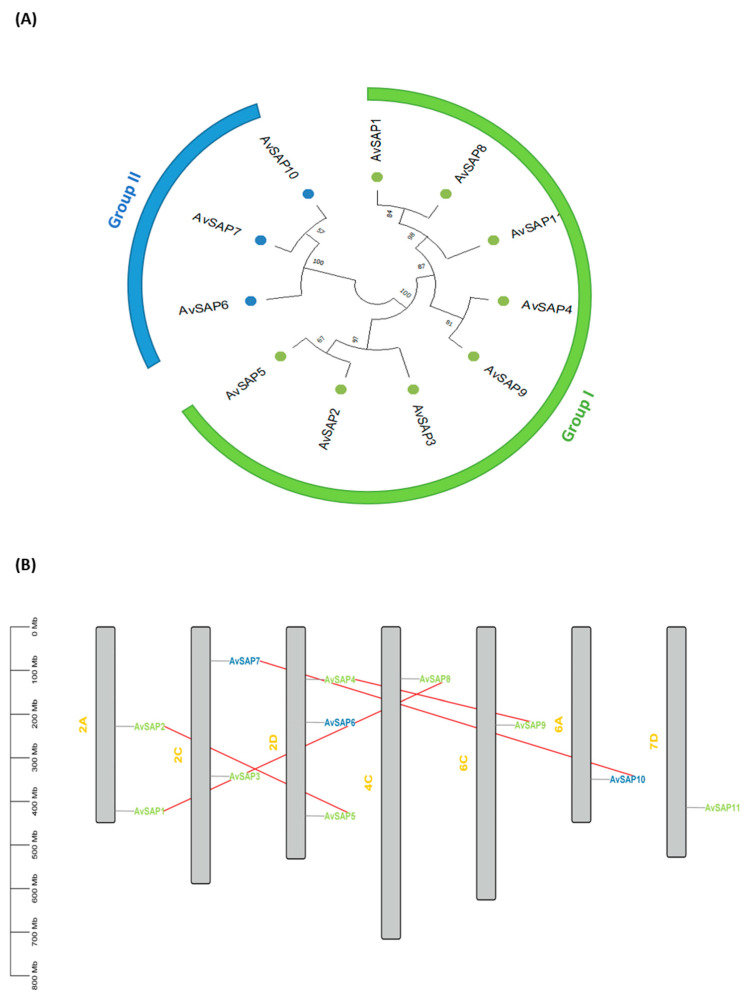
General characteristics of *SAP* genes identified in *Avena sativa* genome. (**A**) Phylogenetic tree of *AvSAP* genes using Mega 11 with maximum likelihood method. (**B**) Chromosomal localization of *AvSAP* genes through the use of the software Tbtools II v2.127. Linked genes pairs are represented by red lines. AvSAP names belonging to phylogenetic group I and II are colored, respectively, with green and blue.

A phylogenetic tree illustrating the relationship between the identified *AvSAP* genes was constructed (Figure 1A). These genes were classified into two major groups. Indeed, the first group comprised eight genes, whereas the remaining genes (*AvSAP6*, *AvSAP7*, and *AvSAP10*) were classified within the second group. In addition, *AvSAP* genes presented a large chromosomic distribution. In fact, these genes were unevenly distributed across the chromosomes, with members exhibiting distinct evolutionary clustering. Overall, *AvSAP* genes were distributed among seven chromosomes. Chromosome 2D harbored three *AvSAP* genes (*AvSAP4*, *AvSAP5*, and *AvSAP6*), whereas both chromosomes 2A and 2C presented two genes (*AvSAP1* and *AvSAP2*, located in Ch 2A, and *AvSAP3* and *AvSAP7*, located in Ch2C). Finally, four *AvSAP* genes (*AvSAP8*, *AvSAP9*, *AvSAP10*, and *AvSAP11*) were present on chromosomes 4C, 6C, 6A, and 7D, respectively (Figure 1B). Four pairs of duplicated genes were identified (*AvSAP1–AvSAP8*, *AvSAP4–AvSAP9*, *AvSAP2–AvSAP5*, and *AvSAP7–AvSAP10*). They are located on different chromosomal regions, suggesting that segmental duplication events played a key role in the expansion of the *AvSAP* gene family (Figure 1B). The calculated ratios of non-synonymous to synonymous substitution (Ka/Ks) for these duplicated pairs proved to be less than 1 (Table 2; Figure 1B), which may be regarded as a purifying selection. Intertwined, these results are indicative that the diversification and the expansion of *AvSAP* genes in the *Avena sativa* genome (OT3098 variety) were mainly triggered by ancient duplication events followed by functional conservation rather than adaptive divergence.

**Figure 2:**
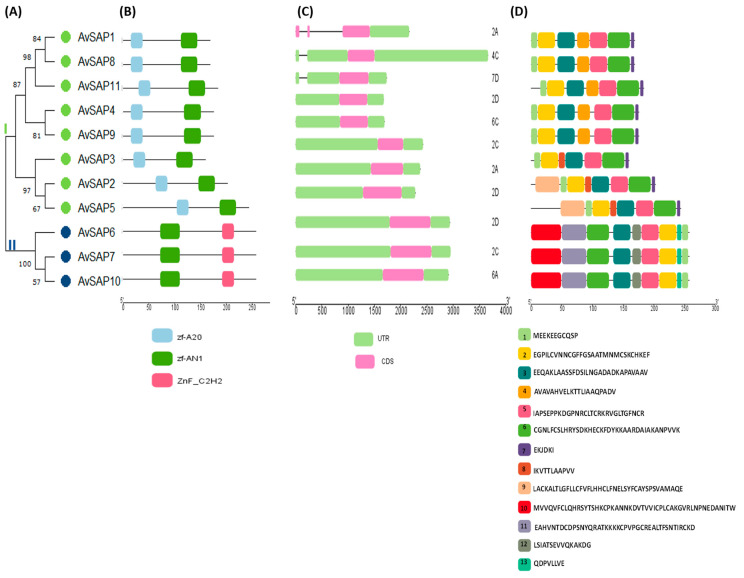
Identification and characterization of *AvSAP* genes/proteins on oats. (**A**) Phylogenetic tree of AvSAP proteins using Mega 11. Saps proteins are divided to two groups I and II. (**B**) Conserved domains of AvSAP visualized by TbTools II v2.127. (**C**) *AvSAP* gene structures using the software Tbtools II v2.127. (**D**) Conserved motif of AvSAP proteins annotated with TbTools II v2.127 software; thirteen motifs are marked with different colors.

The classification of AvSAP proteins into two groups relies upon the presence or absence of conserved domains in their structures (Figure 2A,B). Analysis demonstrated that the AN1 domain was mapped in all identified proteins. This domain was detected in the C-terminal portion of all the proteins except for AvSAP6, AvSAP7, and AvSAP10, where it was located at their N-terminal portion. Notably, the AN20 domain was mapped in eight proteins belonging to the first group (*AvSAP1*, *2*, *3*, *4*, *5*, *8*, *9*, *11*), and it was located at their N-terminal portion. However, AvSAP6, AvSAP7, and AvSAP10 lack the zf-A20 domain and contain the Znf_C2H2 domains at their C-terminal portions, and they are clustered into the second group (Figure 2A,B). Moreover, the analyses of the exon–intron organization were conducted to trace the evolution of the *AvSAP* genes. Analysis revealed that the majority of selected genes share the same structure. Most of those genes (8 out of 11 genes) present one exon, but one gene (*AvSAP1*) presents three exons, whereas *AvSAP8* and *AvSAP11* present two exons (Table 1; Figure 2C). Additionally, *AvSAP1*, *AvSAP8*, and *AvSAP11* genes present introns in their 5′UTR regions (two introns for *AvSAP1* and one intron for *AvSAP8* and *11*, Figure 2C). Further details regarding the positions and characteristic features of these genes are summarized in Table 1.

In addition, these genes are encoded for eleven different proteins with different sizes varying from 167 aa (AvSAP9) to 258 aa (AvSAP6, 7, and 10) in length (Table 3). Four out of the identified proteins are stable, and all SAP proteins are hydrophobic as they present negative GRAVY index. All identified proteins are basic, with isoelectric points varying from 7.48 to 9.18. Their relative molecular weights (MWs) range from 17.43 kDa to 28.55 kDa (Table 3).

Five conserved motifs are present in both *AvSAP* proteins groups: motif 1 (MEEKEEGCQSP), motif 2 (EGPILCVNNCGFFGSAATMNMCSKCHKEF), motif 3 (EEQAKLAASSFDSILNGADADKAPAVAAV), motif 5 (IAPSEPPKDGPNRCLTCRKRVGLTGFNCR), and motif 6 (CGNLFCSLHRYSDKHECKFDYKKAARDAIAKANPVVK) (Figure 2D). Furthermore, a similar arrangement is recorded for both group members. AvSAP2, AvSAP3, and AvSAP5 proteins differ from other proteins by the presence of motif 8 (IKVTTLAAPVV), present uniquely in this subgroup. Moreover, the most closely related proteins AvSAP2 and AvSAP5, which are marked by the presence of motif 9 (LACKALTLGFLLCFVFLHHCLFNELSYFCAYSPSVAMAQE), are detected only in these proteins. Proteins belonging to group II equally possess four specific motifs (motif 10–13). In addition, sequence alignment revealed that *AvSAP* proteins present a high degree of similarity, especially for the conserved domains (Supplemental Figure S1). These findings offer evidence that *AvSAP* family genes are highly conserved in the *Avena sativa* genome and may be involved in various functions.

### 3.2. Phylogenetic Relationship of SAP Proteins

**Figure 3:**
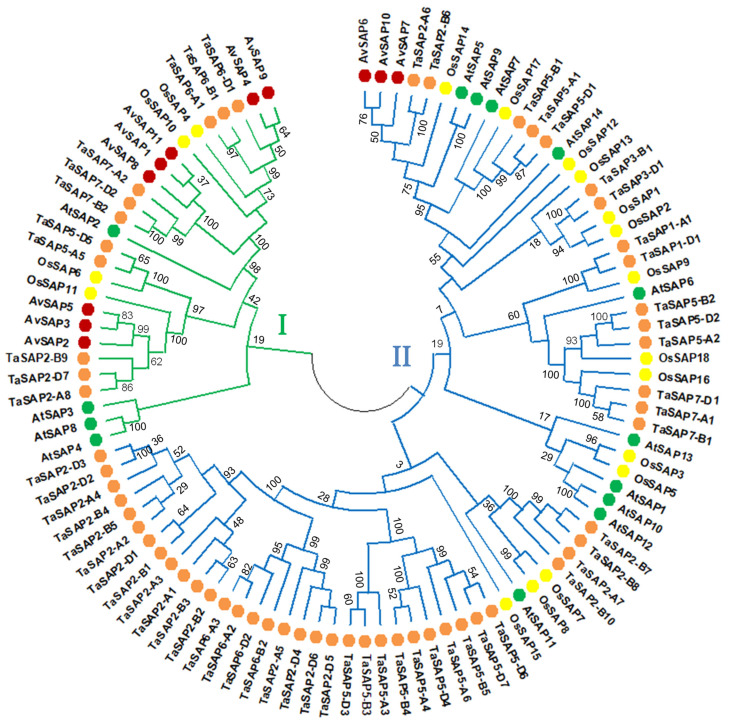
Phylogenetic analysis of putative SAP proteins of *Avena sativa*, *Triticum aestivum*, *Oryza sativa*, and *Arabidopsis thaliana*. The phylogenetic tree is built up using MEGA 11 with SAP protein sequences of 60 *T. aestivum*, 18 of *O. sativa*, 14 *Arabidopsis*, and 11 of AvSAP proteins. The resulting two groups are labeled. SAP from *T. aestivum*, *O. sativa*, *Arabidopsis*, and *A. sativa* are indicated by orange, yellow, green, and red circle shapes, respectively.

To further explore the evolutionary history of the *AvSAP* protein family, we investigated their sequences to build up a phylogenetic tree, including other SAP members from *T. aestivum*, *A. thaliana*, and *O. sativa* (Figure 3). SAP proteins were clustered into two major groups. Group I contains eight out eleven AvSAP proteins. These proteins are characterized by the presence of both domains, A20 and AN1, in their sequences. However, group II includes only three AvSAP and the largest *A. sativa* and *T. aestivum* SAP groups. Obviously, this group involves the highest numbers compared to other classes, which is constituted almost by TaSAP, 14 OsSAP, and 10 AtSAP.

The presence of A20 and AN1 domains across different clades corroborates that these motifs were conserved during evolution due to their crucial role in stress responses. In monocot and dicot plants, SAP proteins are clustered together in the same group (groups II and IV), which may be ascribed to the early divergence of this family across the plant species. Based on this phylogenetic study, we suggest that SAP protein sequences have evolutionarily conserved their functional structural domains over their evolution.

### 3.3. Functional Structure and Subcellular Localization of AvSAP Proteins

**Figure 4:**
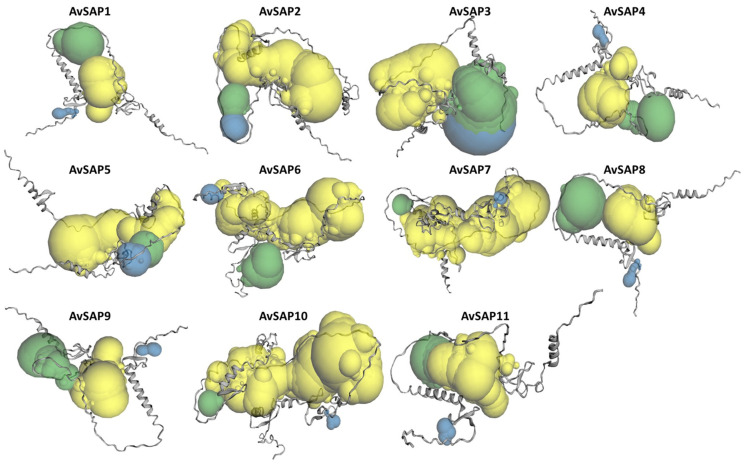
Prediction of AvSAP tertiary structure through the use of the SWISS MODEL online tool. Pockets are visualized from the largest pocket with yellow, green, and blue colors, respectively, by CASTp 3.0.

To investigate the biological function, three dimensional structures of AvSAP were examined. As portrayed in Figure 4, all 3D protein models display similar architectures, which justifies the conservation of AvSAP sequences. Furthermore, three predicted pockets were identified in all tridimensional AvSAP with different volumes. These results are conductive to the possibility of multiple ligand interactions with AvSAP proteins in *A. sativa* plants. Moreover, the prediction of subcellular localizations of these proteins indicates that the AvSAP family proteins are located in different compartments (Supplementary Figure S2). Indeed, three proteins are nuclear (AvSAP6, AvSAP7, and AvSAP10). AvSAP10 is also present in peroxisome in addition to AvSAP2, AvSAP3, and AvSAP5. However, proteins AvSAP1, 8, and 11 prove to be cytoplasmic. AvSAP1 and AvSAP9 proteins may be located in extracellular space. Some proteins appear also in the chloroplast, especially AvSAP4 which had a significant prediction to be in this organelle.

### 3.4. Analysis of A. sativa SAP Promoter Sequence

**Figure 5:**
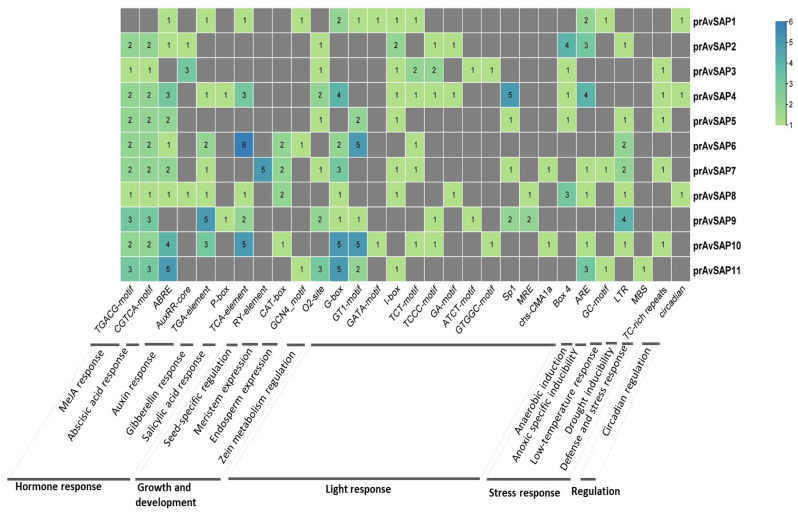
Prediction of the putative cis-acting elements number of AvSAP promoter regions by means of the PlantCare online tool.

A myriad of cis-regulatory elements (CREs) identified in SAP promoters were classified into four groups related to stress response, growth and development, regulation, and hormone response (Figure 5). All AvSAP promoters’ regions possess MeJA response cis-elements (CGTCA, TGACG), except for AvSAP1, in addition to CREs involved in gibberellin, auxin, abscisic acid, and salicylic acid. In fact, six cis-regulatory elements of salicylic acid response (TCA-element) are mapped in prAvSAP6, corresponding to the highest putative number predicted in all promoters. In addition, 13 motifs involved in light-responsiveness are found in AvSAP promoters with different combinations. For instance, five prAvSAP4 harbors Sp1, four G-box, and five other motifs (I-box, TCT-motif, TCCC-motif, GA-motif, and Box4). Furthermore, supplementary cis-element functions associated with growth and development elements are found in AvSAP promoters, such as seed-specific regulation, meristem expression, endosperm expression, and metabolism regulation. CRE in prAvSAP is equally involved in stress response and is present as an ARE motif (anaerobic response element), an LTR (low-temperature response element), an MBS (drought response), a TC-rich repeat (defense and stress responsiveness), and a GC-motif (anoxic specific inducibility). ARE is found in almost all promoters, where four motifs are predicted in prAvSAP4 and three motifs in both prAvSAP2 and prAvSAP11. The latter proves to be the only drought-responsive element (MBS). PrAvSAP is also implicated in circadian regulation. These findings are indicative that AvSAP promoters may be involved in plant defense and play crucial roles in gene regulation.

### 3.5. QRT-PCR Analysis of AvSAP Genes

**Figure 6:**
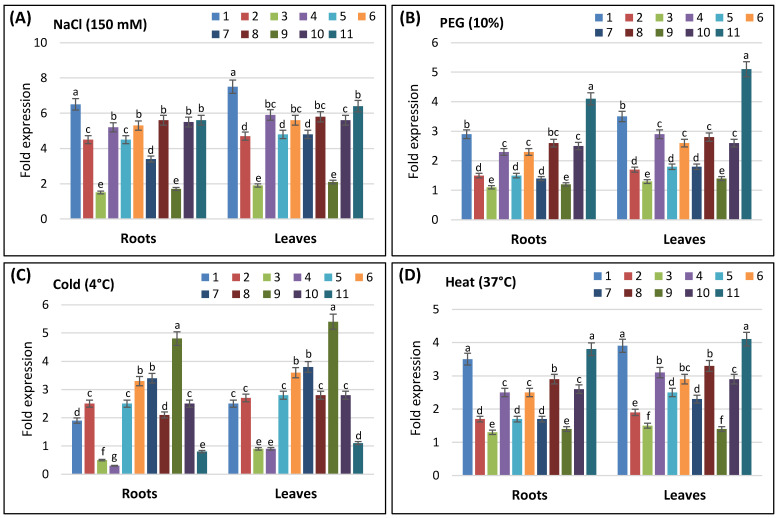
Analyses of the expression profiles of *AvSAP* genes in leaves and roots of *A. sativa* exposed to abiotic stress for 48 h. Two-week-old plants were treated with 150 mM NaCl (**A**), 10% PEG (**B**), cold stress (4 °C) (**C**), and heat stress (37 °C) (**D**). The expression value of each *AvSAP* gene in leaves and roots in the non-treated plants (control) was set as 1 in order to calculate the relative expression. Subsequently, log_2_ transformed values are presented in bar charts. *GAPDH* and *ADP-ribosyl cyclase* (*ADPR*) genes were used as internal controls. Four plants were used per treatment per replicate. Error bars indicate standard deviation of three biological replicates. Different letters marked on the same bar chart denote significant differences (*p* < 0.05), as evidenced by a two-way ANOVA test.

In order to investigate the response of *AvSAP* genes to external stimuli, the expression profiles of identified genes were studied using QRT-PCR. In response to NaCl, all *AvSAP* genes were highly upregulated in roots and shoots after 48 h of stress application except for *AvSAP3* and *AvSAP9*, which presented a slight upregulation, especially in roots (Figure 6A). *AvSAP1* was the most upregulated genes with the most fold inductions between the identified genes. The drought stress, mimicked by PEG (15%) treatment, caused the upregulation of all *AvSAP* genes. Further analysis demonstrated that *AvSAP3* and *AvSAP9* presented a slight upregulation, whereas *AvSAP1*, *AvSAP4*, *AvSAP6*, and, especially, *AvSAP11* presented the highest fold inductions. These results confirm that *AvSAP* genes may be important regulators of plants’ responses to osmotic stress (Figure 6B).

Under cold stress, *AvSAP3*, *AvSAP4*, and *AvSAP11* were not upregulated, whereas the remaining genes, especially *AvSAP9*, were upregulated (Figure 6C). In contrast, all *AvSAP* genes were upregulated in oats in response to heat stress (Figure 6D).

**Figure 7:**
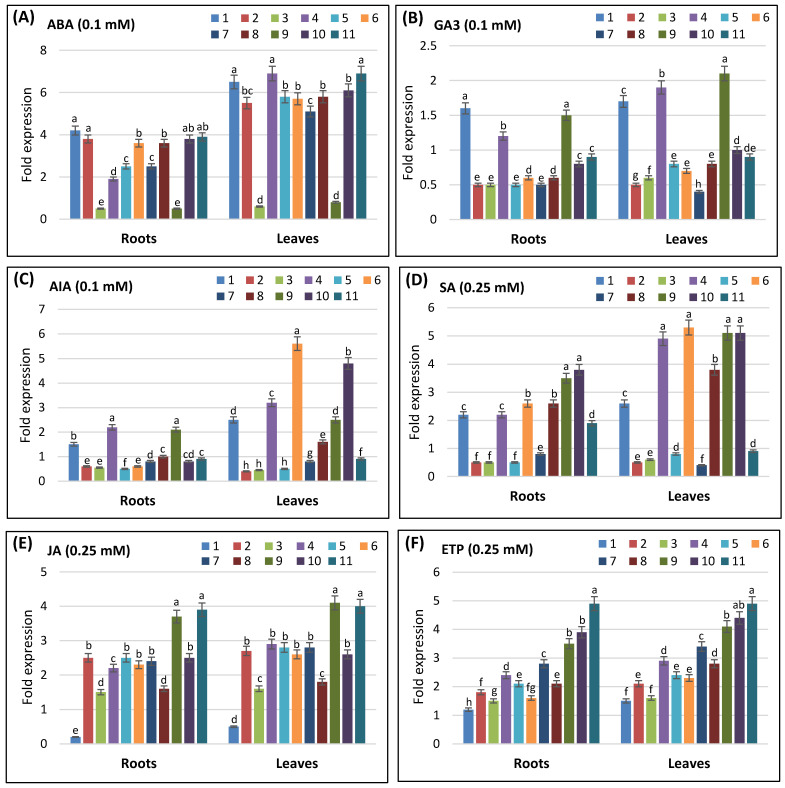
Analyses of expression profiles of *AvSAP* genes in leaves and roots of *A. sativa* exposed to hormonal stress for 48 h. Two-week-old plants were treated with 0.1 mM ABA (**A**), 0.1 mM GA3 (**B**), 0.1 mM AIA (**C**), 0.25 mM SA (**D**), 0.25 mM JA (**E**), and 0.25 mM ETP (**F**). The expression value of each *AvSAP* gene in leaves and roots in the non-treated plants (control) was set as 1 in order to calculate the relative expression. Next log_2_ transformed values are presented in bar charts. *GAPDH* and *ADP-ribosyl cyclase* (*ADPR*) genes were used as internal controls. Four plants were used per treatment per replicate. Error bars indicate standard deviation of three biological replicates. Different letters marked on the same bar chart indicate significant differences (*p* < 0.05), as proved by a two-way ANOVA test.

In response to hormonal stress, ABA exerted a similar effect (Figure 7A) to that observed under osmotic stress. All *AvSAP* genes were upregulated in response to ABA, except for *AvSAP3* and *AvSAP9*, with a more pronounced upregulation in leaves compared to roots. In response to GA3, only four genes were upregulated: *AvSAP1*, *AvSAP4*, and *AvSAP9*. Moreover, in response to AIA, *AvSAP1*, *AvSAP4*, and *AvSAP9* genes were upregulated in roots, whereas in leaves, such genes as *AvSAP6*, *AvSAP8*, *AvSAP 10*, and *AvSAP11* were also upregulated (Figure 7B,C).

SA stress was equal. This hormone triggered the upregulation of *AvSAP1*, *AvSAP4*, *AvSAP6*, *AvSAP8*, *AvSAP10*, and *AvSAP11* genes in roots with a better fold induction in leaves (Figure 7D). On the other side, JA induced the upregulation of all *AvSAP* genes except for *AvSAP1* (Figure 7E). Finally, Etephon was also used, revealing that this element yielded the upregulation of all *AvSAP* genes (Figure 7F). All these data suggest that *AvSAP* genes can play crucial roles in oat response to various phytohormones.

**Figure 8:**
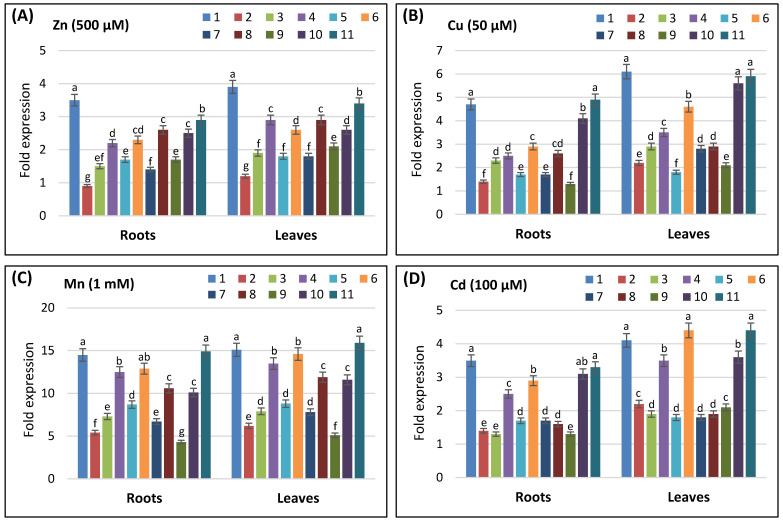
Analyses of the expression profiles of *AvSAP* genes in leaves and roots of *A. sativa* exposed to various heavy metal stress for 48 h. Two-week-old plants were treated with 500 µM ZnSO_4_ (**A**), 50 µM CuSO_4_ (**B**), 1 mM MnCl_2_ (**C**), and 100 µM CdCl_2_ (**D**). The expression value of each *AvSAP* gene in leaves and roots in the non-treated plants (control) was set as 1 in order to calculate the relative expression. The log_2_ transformed values are presented in bar charts. *GAPDH* and *ADP-ribosyl cyclase* (*ADPR*) genes were used as internal controls. Four plants were used per treatment per replicate. Error bars indicate standard deviation of three biological replicates. Different letters marked on the same bar chart denote significant differences (*p* < 0.05), as corroborated by a two-way ANOVA test.

Finally, the role of *AvSAP* genes in response to heavy metal stress was also examined. Four different heavy metals were selected, namely, Zn, Cu, Mn, and Cd (Figure 8). Our analysis demonstrated that all *AvSAP* genes were also responsive to these elements. Basically, our analysis allows for the first comprehensive description of *SAP* genes in oat.

### 3.6. Recombinant AvSAP1 Proteins Confer Bacterial Tolerance to Ionic and Osmotic Stress

**Figure 9:**
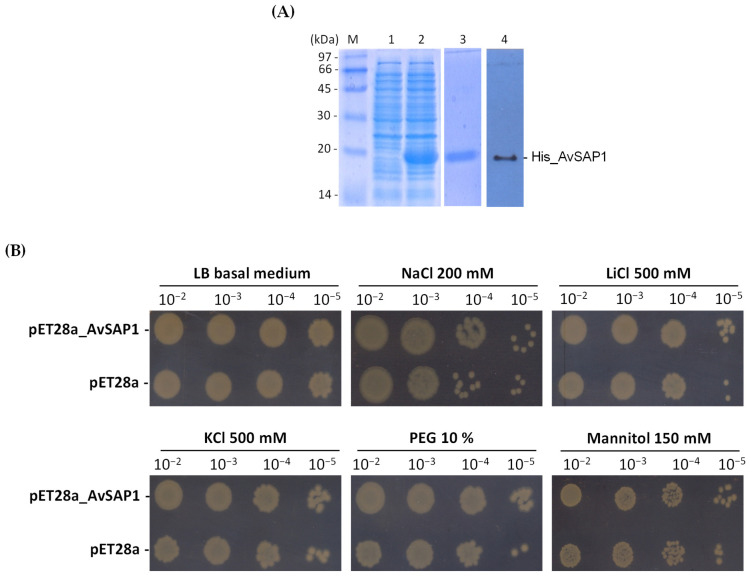
Analysis of the protective role of *AvSAP1* from various stresses in vitro. (**A**) SDS-PAGE analyses of the purified recombinant protein His_AvSAP1. (**B**) Behavior of AvSAP1 transformed bacteria in solid LB medium in the presence or absence of abiotic stress. *E. coli* cells transformed with the empty vector (pET28a) or with the recombinant vectors (pET28a + AvSAP1) were grown for 24 h under normal growth conditions or stressed mediums.

As the *AvSAP1* gene was responsive to all tested abiotic and metallic stresses and to the majority of hormonal stresses, its heterologous expression in *E. coli* cells served to examine the biological function of *AvSAP1* in the tolerance to abiotic stress in vivo. After being cloned in the pET128a expression vector, AvSAP1 full-length cDNA was introduced into the BL21 strain of *E. coli* and then purified the AvSAP1 protein. The expression of AvSAP1 was obtained 6 h after the addition of 1 mM IPTG. At this level, we observed an intense protein band on SDS-PAGE with a molecular weight (MW) of about 20 kDa, corresponding to the estimated MW of the AvSAP1 protein (Figure 9A). To check whether this band corresponds to the purified His-tagged *AvSAP1* or not, we conducted a Western blot analysis using the anti-6x-His tag monoclonal antibody (Invitrogen) as a primary antibody. The immunoblot analysis revealed a signal having the same MW as the purified His-tagged *AvSAP1* protein (Figure 9A).

To assess the biological role of the full-length *AvSAP1* cDNA in response to abiotic stress, the growth of transformed *E. coli* cells transformed with *AvSAP1* or the empty vector was evaluated under various stresses (200 mM NaCl, 0.5 M LiCl, 500 mM KCl, 10% PEG6000, and 150 mM mannitol) in solid media. All examined strains were developed similarly on solid media and did not exhibit any discernible differences in growth under regular circumstances (Figure 9B). In contrast with all stress treatments, cells transformed with *AvSAP1* recombinant plasmids displayed greater growth rates than cells transformed with empty vector (Figure 9B). These findings prove that *AvSAP1* expression can play a positive role in regulating *E. coli* cell growth under a variety of stress situations.

## 4. Discussion

Over the last two decades, several research works have been particularly oriented toward investigating the role of A20/AN1 domain containing proteins, known as the stress-associated protein (SAP) family. In this respect, these proteins display structural and functional preservation among plant species [4,5]. In plants, different studies have reported that *SAP* genes are induced by at least one abiotic stress and function in a stress- and/or tissue-specific manner [5,22,43,44]. Within this context, numerous analyses have highlighted that SAP proteins act as redox sensors, ubiquitin ligases, and/or gene expression regulators [4,44,45,46,47]. Moreover, these proteins are involved in plant response to various stress conditions [12,21,29,48].

Exploring SAP biological functions has become a topic of considerable interest and ongoing research as scientists seek deeper insights into plant stress responses. *SAP* genes are multigenic families and have been identified in different plants such as in *T. aestivum*, *Ae. tauschii*, *T. dicoccoides* and *T. urartu* [25], cotton (*Gossypium hirsutum*) [24], *M. truncatula* [23], apple [13], sunflower (*Helianthus annuus*) [26], *O. sativa*, and *Arabidopsis thaliana* [10]. This motivated us to pursue this specific path. Indeed, in silico genome-wide analysis revealed the presence of 11 *AvSAP* genes in the *Avena sativa* (OT3098 variety) genome. The structure of the SAP proteins is known by harboring a ZF-AN1 domain at their C-terminus defined by a [Cys-X2-Cys-X(9-12)-Cys-X(1-2)-Cys-X4-Cys-X2-His-X5-His-X-Cys] signature and/or a ZF-A20 zinc-finger domain [Cys-X(2-4)-Cys-X11-Cys-X2-Cys] at their N-terminus. Basically, C2H2 domains can also be present in SAP protein structures [49]. In fact, as depicted in Figure 2B, three AvSAP proteins exhibit C2H2 domains in addition to ZF-AN1 domains, while the other AvSAP proteins are present as a classic SAP type possessing ZF-AN1 and ZF-A20 zinc-finger domains. Previous studies indicated that most of the SAP families exhibit AN1 and A20 zinc-finger domains. The majority of SAP families are composed of proteins harboring the AN1 domain and retaining those with only A20 domains in several plants such as sunflower [26], maize [21], *M. truncatula* [23], potato [28], and wheat [25]. Yet, a few SAP proteins are characterized as the A20-type; 5 out of 57 in *Brassica napus* [50], 1 out of 15 in grapevine [51], and 3 out of 30 in apple [13]. Proceeding in the same way as the findings recorded in prior works, these results indicate the high conservation of the AN1 domain among SAP proteins, and suggest that this domain is evolutionarily older than the A20 domain [10,26].

To confirm the conservation of SAP structures during their evolution, we constructed a phylogenetic tree. SAP proteins from different plant species were classified into two groups. In this respect, AvSAP without A20 domains contain AN1 and C2H2 domains (AvSAP6, 7, and 10) belonging to group II (blue color branch) with SAP proteins possessing two AN1 domains and two C2H2 domains from *T. aestivum* (TaSAP2-A6, TaSAP2-B6), *A. thaliana* (AtSAP5 and AtSAP9), and *O. sativa* (OsSAP14). The other *Avena sativa* SAP proteins, AtSAP1, 2, 3, 4, 8, 10, 12, and OsSAP4, 6, 10, and 11 and all TaSAP (except TaSAP2-D5, which contains AN1 and C2H2 domains) include A20 and AN1 domains and are present in group I (green color branch). These findings are indicative of the high conservation of SAP sequences during their phylogenetic evolution.

Furthermore, the arrangement of exon–intron in architecture is also involved in the evolution of the gene family. In this line, the analysis of *AvSAP* gene structures reveals the high similarity among their nucleotide sequences. While the majority of *AvSAP* genes are intron-free, three of them possess one (*AvSAP8* and *AvSAP11*) or two (*AvSAP1*) introns. Our results are in good accordance with the previous findings, where most genes in other plants are present as intron-free genes. For instance, in sunflower [26], apple [13], rice, and *Arabidopsis thaliana* [10], approximately 60 to 70% of *SAP* genes lack introns, 20 to 30% present a single intron, and only 10% of *SAP* plant genes possess two introns. The lack of introns is a pivotal characteristic of the *SAP* gene family [13,26]. However, there are some exceptions rarely observed in SAP plants, as detected for MtSAP13, presenting four introns [23]. Possessing an intron-free gene is accounted for in terms of the earlier origin of the SAP family and the rapid protein translation by the reduction in the post-transcriptional process under stresses [52]. As far as the current research work is concerned, the evolution of the *SAP* gene family is also assigned to the presence of the gene duplications. The homologies among the *AvSAP* gene members reflect their similar biological functions. The cis-elements are present in *AvSAP* promoter regions with variability of numbers and functions, which suggests that *AvSAP* genes can be regulated upon different stresses.

Different reports assert that *SAP* genes are involved in plant responses to different environmental stresses [21,29,53]. At this stage, we have demonstrated that different *AvSAP* genes promoters present different stress-responsive cis-elements, indicating that the *AvSAP* genes can participate in plant responses to different environmental stresses.

The presence of several hormones and stress-responsive cis-elements in the *AvSAP* promoters matches their activation during various environmental stresses. In particular, the presence of ABRE (abscisic acid (ABA)), TGACG, and CGTCA (methyl jasmonate (MeJA)) cis-elements in their promotors (Figure 5) aligns with the significant upregulation of most *AvSAP* genes (*AvSAP1*, *AvSAP4*, *AvSAP6*, and *AvSAP11*) after ABA and JA treatments (Figure 7A,E). Similarly, the presence of TCA-elements, which relates to salicylic acid (SA), supports the activation of *AvSAP4*, *AvSAP6*, *AvSAP8*, *AvSAP10*, and *AvSAP11* after SA treatment (Figure 7C). In contrast, *AvSAP3* and *AvSAP9*, ABRE elements, are absent in their promotor sequences, displaying downregulation after ABA treatments. Additionally, finding MBS- and TC-rich repeat motifs (Figure 5), notably linked to drought and defense responses, might account for the higher expression of *AvSAP1*, *AvSAP4*, *AvSAP6*, and *AvSAP11* under PEG-induced osmotic stress (Figure 6B). Basically, the link between cis-element enrichment and expression profiles offers evidence for the complex control of *AvSAP* genes and supports their role in hormonal and abiotic stress adaptation in oats.

RT-PCR analysis demonstrated that all *AvSAP* genes were upregulated in response to osmotic stress. The same result was also detected in maize [21]. In cucumber, *CsSAP5*, *CsSAP6*, *CsSAP9*, and *CsSAP10* proved to be induced in response to salt, drought, and osmotic stresses [29]. On the other side, we found that *SAP* genes were responsive to the heavy metal stresses. In fact, *AvSAP1*, *AvSAP10*, and *AvSAP11* genes were strongly upregulated in response to all tested heavy metals, whereas *AvSAP2* was responsive to Mn stress and slightly to Cu and Cd, but not Zn. The remaining genes were slightly responsive to these stresses.

Different research works have extensively revealed that *E. coli* cell growth can be modulated under various stress-expressing recombinant proteins [54,55,56,57]. Thus, to gain insight into the function of *AvSAP1*, we first expressed it in *E. coli* BL21 cells, and then His-tagged AvSAP1 protein was purified. Protein targeting His tail (6-His) facilitates the recombined protein elution using a gradient concentration of imidazole and thereby facilitating proteins purification. Accordingly, it is inferred, departing from the above analysis, that *AvSAP1* conferred a tolerance phenotype of the transformed *E. coli* cells to salt, ionic, and osmotic stresses (Figure 9B), suggesting that *AvSAP1* is a functional gene and plays a positive key role in response to various abiotic stresses in *E. coli* cells. Recently, it has been confirmed that heterologous expression of *ZmSAP2* and *ZmSAP7* genes, but not the other identified *ZmSAP* genes, promotes yeast tolerance to high salt and not mannitol. Interestingly, the relative expression of both genes is reduced by salt in maize [58]. Moreover, Ben Saad et al. [11,43] emphasized that heterologous expression of the *SAP* gene from *Aeluropus littoralis* (*AlSAP*) and *Lobularia maritima* (*LmSAP*) equally fosters cell tolerance to salt, ionic, and osmotic stresses in yeast. In addition, *Leymus chinensis* (*LcSAP*) boosts bacterial survival rate under different abiotic stresses [53].

## 5. Conclusions

Toward the conclusion of the present study, we assert that our work provides the first genome-wide analysis of the *SAP* gene family in oat (OT3098 variety). A total of eleven *AvSAP* genes were mapped and analyzed regarding their localization, structure, conserved domain organization, phylogenetic relationships, and tissue-specific expression patterns. This study allows a deeper dive into these gene members in oat, and, thus, into their encoded proteins. Relying on a thorough investigation of the eleven *AvSAP* gene expression profiles under abiotic stress conditions, novel information was acquired concerning the role of *SAP* genes in oat’s response to environmental stimuli. As a final note, it is noteworthy that in vitro *AvSAP1* proved to be a functional gene in the *E. coli* system that is able to impact abiotic stress tolerance in bacteria. The generation of transgenic cereal crops by the overexpression of *AvSAP* genes may, therefore, improve their stress tolerance and support climate-resilient agriculture. These findings may be regarded as valuable and promising, as they may serve as an enlightening guideline and theoretical background for improving stress tolerance in oats and other crop plants.

## Figures and Tables

**Table 1:** Genomic features of *AvSAP* genes.

	Transcript ID	Chr Position Start…End	Strand	Transcript Length (bp)	Number of Exons
*AvSAP1*	AVESA.00001b.r3.2Ag0002549.1	Chr 2A: 421,880,149–421,882,310	Forward	1385	3
*AvSAP2*	AVESA.00001b.r3.2Ag0000617.3	Chr 2A: 227,690,788–227,693,159	Forward	2372	1
*AvSAP3*	AVESA.00001b.r3.2Cg0001305.2	Chr 2C: 342,089,875–342,092,300	Forward	2426	1
*AvSAP4*	AVESA.00001b.r3.2Dg0001165.3	Chr 2D: 120,505,420–120,507,098	reverse	1679	1
*AvSAP5*	AVESA.00001b.r3.2Dg0003154.1	Chr2D: 433,132,357–433,134,635	forward	2279	1
*AvSAP6*	AVESA.00001b.r3.2Dg0002493.2	Chr 2D: 218,475,273–218,478,209	reverse	2937	1
*AvSAP7*	AVESA.00001b.r3.2Cg0000502.2	Chr 2C: 78,030,240–78,033,190	forward	2951	1
*AvSAP8*	AVESA.00001b.r3.4Cg0001460.1	Chr 4C: 119,123,905–119,127,569	reverse	3510	2
*AvSAP9*	AVESA.00001b.r3.6Cg0002050.3	Chr6C: 224,744,555–224,746,247	reverse	1693	1
*AvSAP10*	AVESA.00001b.r3.6Ag0002278.3	Chr 6A: 349,101,741–349,104,652	forward	2912	1
*AvSAP11*	AVESA.00001b.r3.7Dg0002162.2	Chr 7D: 414,026,376–414,028,110	forward	1582	2

**Table 2:** Ka/Ks values for the estimated segmental duplication of *AvSAP* genes.

Gene_1	Gene_2	Ka	Ks	Ka_Ks
*AvSAP1*	*AvSAP8*	0.005194825963401796	0.20888707907827586	0.024869063162375634
*AvSAP4*	*AvSAP9*	0.009876685942230679	0.13632577018800407	0.07244914830563542
*AvSAP2*	*AvSAP5*	0.02140176385683392	0.02996729652881873	0.7141706572113514
*AvSAP7*	*AvSAP10*	0.01593128653818524	0.07675403492168237	0.20756285391955054

**Table 3:** Physicochemical characterization of AvSAP proteins, as obtained using the protparam online tool.

Protein Name	Protein Length (aa)	Molecular Weight (Da)	Theoretical pI	The Instability Index (II)	Aliphatic Index	Gravy
AvSAP1	169	17,945.6	8.63	25.77	70.06	−0.228
AvSAP2	203	22,244.64	7.89	48.63	69.26	−0.095
AvSAP3	160	17,430.90	8.32	49.78	56.19	−0.431
AvSAP4	176	18,850.65	8.28	25.93	68.69	−0.244
AvSAP5	244	26,913.27	7.84	46.74	77.17	0.060
AvSAP6	258	28,395.16	9.04	44.28	64.96	−0.566
AvSAP7	258	28,475.26	8.96	50.34	63.45	−0.571
AvSAP8	169	17,962.65	8.76	25.60	67.75	−0.239
AvSAP9	167	18,767.52	8.28	27.96	66.53	−0.266
AvSAP10	258	28,551.35	9.18	45.42	63.45	−0.617
AvSAP11	184	19,559.59	8.55	25.34	72.28	−0.116

## Data Availability

The data are contained within the article and the Supplementary Materials.

## Supplementary Materials

The following supporting information can be downloaded at: https://www.mdpi.com/article/10.3390/life16010046/s1. Figure S1: Alignment of AvSAP protein sequences by using the MUSCLE algorithm of MEGA 11 and visualized by GeneDoc software. Conserved residues are highlighted by blue rectangle. Conserved A20 and AN1 domains are marked by blue and green boxes respectively; Figure S2: Subcellular localization of the AvSAP proteins using the WoLF PSORT online tool; Table S1: Evaluation of built 3D-protein structures by ProSA-web online tool; Table S2: List of primers used in QRT-PCR analysis; Table S3: List of primers used in molecular cloning of *AvSAP1* gene.
