# Supplementary material for: The SAP Gene Family in Oat (Avena sativa L.): Genome-Wide Identification, Gene Expression Analysis, and Functional Characterization of AvSAP1 in Response to Stress Conditions
Source: Life (Basel). 2025 Dec 26;16(1):46. doi: 10.3390/life16010046 (PMC12842820; doi:10.3390/life16010046)
Supplement: Supplementary file 1 [file life-16-00046-s001.zip › life-4040529-supplementary.pdf]

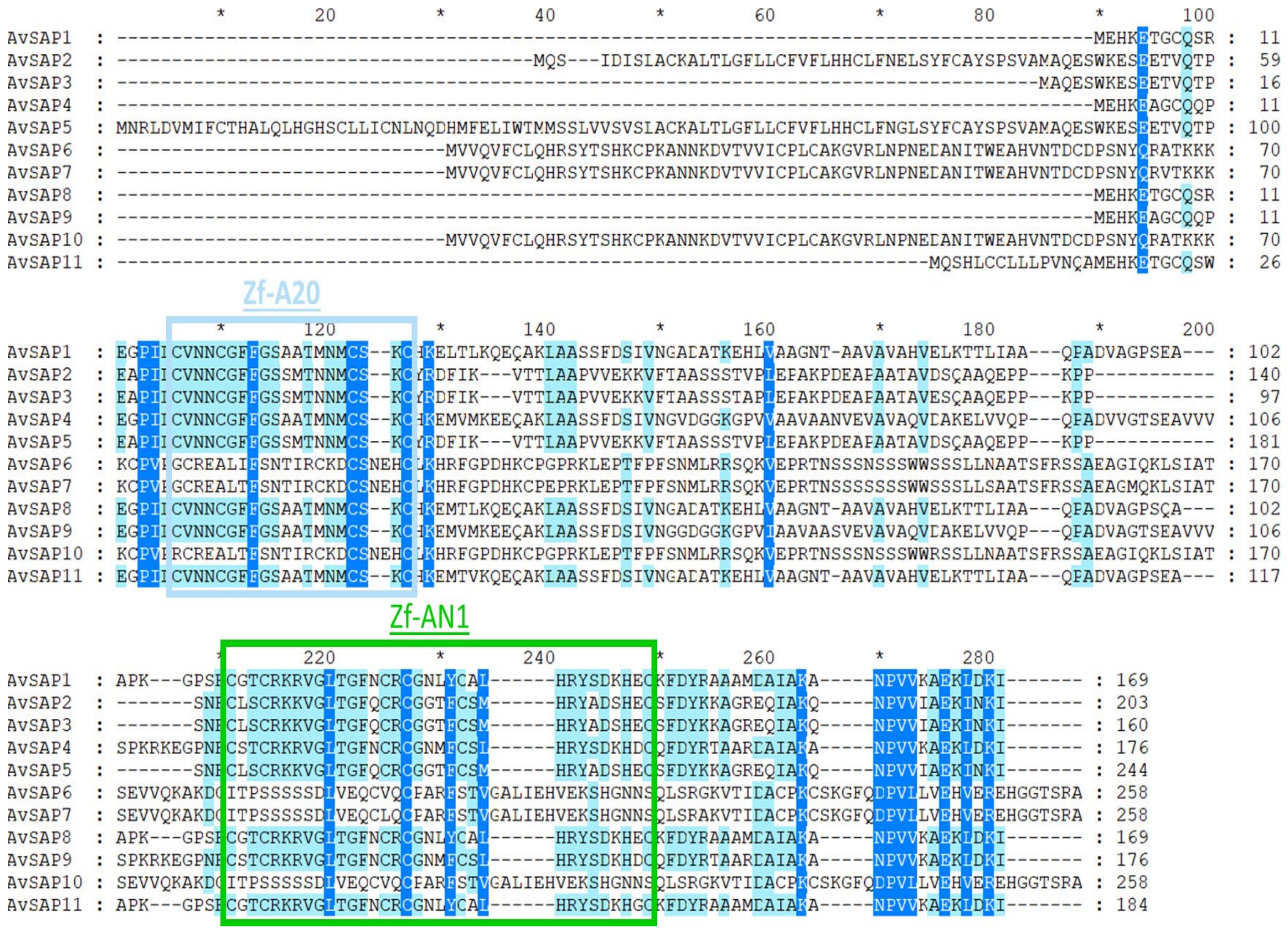

Figure S1

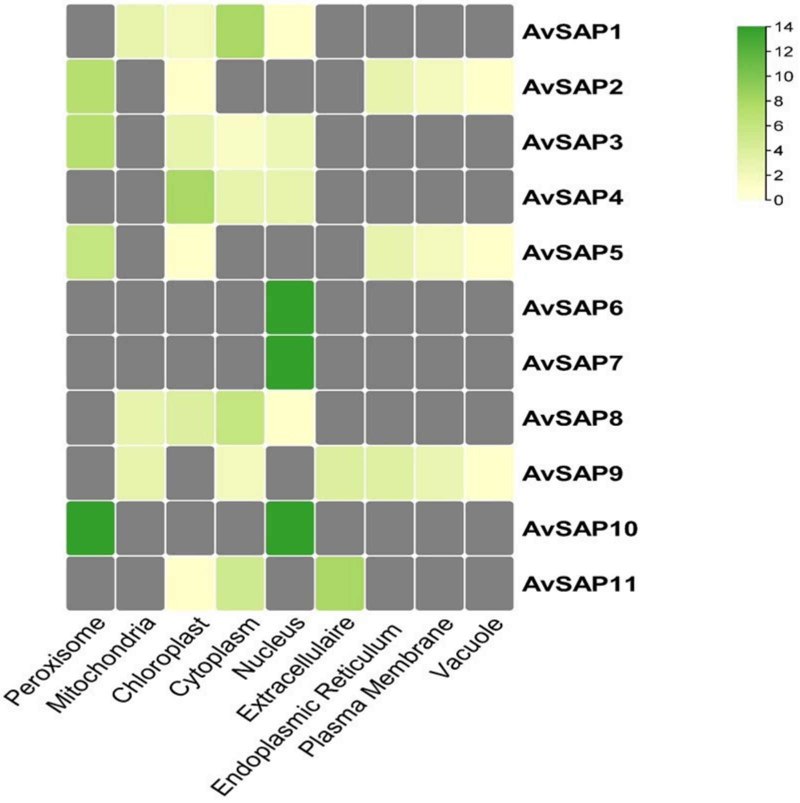
AvSAP1
AvSAP2
AvSAP3
AvSAP4
AvSAP5
AvSAP6
AvSAP7
AvSAP8
AvSAP9
AvSAP10
AvSAP11
Peroxisome
Mitochondria
Chloroplast
Cytoplasm
Nucleus
Extracellulaire
Endoplasmic Reticulum
Plasma Membrane
Vacuole
14
12
10
8
6
4
2
0

Figure S2

Table S1

Protein ID	Z-Score
AvSAP1	-3.49
AvSAP2	-2.69
AvSAP3	-3.49
AvSAP4	-4.34
AvSAP5	-2.46
AvSAP6	-375
AvSAP7	-3.82
AvSAP8	-3.38
AvSAP9	-4.18
AvSAP10	-3.68
AvSAP11	-2.75

Table S2

Gene name	Forward primer	Reverse primer
AvSAP1	CGAGCTGAAGACGACACTTATT	TAAGCCCGACCCTCTTTCT
AvSAP2	CCCTGTAGTGGAGAAGAAAGTA	CTTGGCTATCAACCGCAGTA
AvSAP3	CCCTGTAGTGGAGAAGAAAGTA	GTGCTGCTTGGCTTTCAAC
AvSAP4	CTACTTGCAGGAAGAGGGTTG	GCAGTCATGCTTGTCGGAATA
AvSAP5	CTGAGGAGACTGTCCAAACAC	CTGTAGCACTTCGAGCACAT
AvSAP6	CCTTCAGCACCGAAGTTATACA	CAGACGAACTCCTTTAGCACATA
AvSAP7	ATTGAGCCGTGCCAAAGT	TCCTCCATGTTCCCTCTCAA
AvSAP8	CCACAAGGAGATGACACTGAA	GTTACCAGCAGCAACAAGATG
AvSAP9	TGGGAAAGGACCTGTGATTG	AACATCGGCAGGTTGAGG
AvSAP10	GAAAGTTGAGCCACGTACAAAC	GTATTCCAGCTTCCGCTGAT
AvSAP11	CACAAGGAGATGACGGTGAAG	TGCCAGCAGCAACAAGAT
GADPH	GTTTGGCATCGTTGAGGGTT	TGCTGCTGGGAATGATGTTG
ADPR	CTCATGGTTGGTCTCGATGC	ACATCCCAAACAGTGAAGCT

Table S3

Primer name	Primer sequence	Utilization
AvSAP1_Fr	ATGGAGCACAAGGAAACCGGCT	pGEMT Easy clonage
AvSAP1_Rv	GATCTTGTCGAGCTTCTCCGCT	
His-AvSAP1_Fr	GAATTCATGGAGCACAAGGAAACCGGCT	pET28a clonage
His-AvSAP1_Rv	GCGGCCGCGATCTTGTCGAGCTTCTCCGCT	
